# Assessment of Essential Nutrients, Bioactive Compounds, and Antioxidant Activity in the Leaves and Fruits of Chinese Olive ( *Canarium album* (Lour.) DC.) Cultivars

**DOI:** 10.1002/fsn3.71049

**Published:** 2025-10-24

**Authors:** Qingli Zhuang, Xin Tan, Peiwen Ma, Duo Lai, Xuehua Shao, Ruilian Lai, Weiqiang Xiao

**Affiliations:** ^1^ Institute of Fruit Tree Research Guangdong Academy of Agricultural Sciences; Key Laboratory of South Subtropical Fruit Biology and Genetic Resource Utilization, Ministry of Agriculture and Rural Affairs; Guangdong Provincial Key Laboratory of Science and Technology Research on Fruit Tree Guangzhou China; ^2^ Department of Gastroenterology Beijing Puren Hospital Beijing China; ^3^ Fruit Research Institute Fujian Academy of Agricultural Sciences Fuzhou Fujian China

**Keywords:** antioxidant capacity, bioactive compound, *Canarium album*, essential nutrient element

## Abstract

In this study, leaves and fruits from 13 major Chinese olive ( *Canarium album* (Lour.) DC.) cultivars were analyzed to identify cultivars with high medicinal value, as indicated by their profiles of essential nutrients, bioactive compounds, and antioxidant activity. Significant genotypic variations were observed among cultivars. Correlation analysis revealed leaf calcium and nitrogen negatively impacted flavonoid accumulation, while fruit calcium enhanced phenolics and alkaloids synthesis. In addition, flavonoids were found to be the dominant drivers of antioxidant activity. Notably, Principal component analysis (PCA) and hierarchical cluster analysis (HCA) revealed that the Suanghuxiang, Liehuo, and Zachi cultivars exhibited the highest total flavonoid content and antioxidant capacity in leaves, while Xiaodingxiang, Wangbo, Fengyu1, Ma2, and Langshang showed the highest levels of these properties in fruits. Conversely, the Fenghu, Xi1, Tianzhong, Lingfeng, and Qingying cultivars displayed the lowest overall medicinal properties, characterized by the lowest levels of total flavonoid content and antioxidant activity. This study identifies elite cultivars for medicinal use and provides a foundation for breeding programs and optimized fertilization.

## Graphical abstract

Key Findings: Analysis of 13 Chinese olive cultivars revealed significant genotypic variations in their profiles of essential nutrients, bioactive compounds, and antioxidant activity. Correlation analysis showed leaf calcium/nitrogen reduced flavonoid accumulation, while fruit calcium enhanced phenolics/alkaloids synthesis, with flavonoids identified as the dominant drivers of antioxidant activity. PCA and HCA revealed that leaves of Suanghuxiang, Liehuo, Zachi and fruits of Xiaodingxiang, Wangbo, Fengyu1, Ma2, Langshang showed the highest flavonoid/antioxidant levels, whereas Fenghu, Xi1, Tianzhong, Lingfeng, Qingying had the lowest.

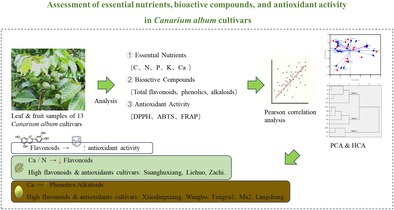

## 1 Introduction

The Chinese olive ( *Canarium album* (Lour), DC.), locally known as “Ganlan,” is a prominent fruit tree belonging to the Burseraceae family, widely cultivated in southern China and Southeast Asia (Kuo et al. 2016). It is well established that both leaves and fruits of the Chinese olive exhibit multiple pharmacological properties, including strong antioxidant activity (Zhang and Lin 2008; Chang et al. 2017), anti‐inflammatory effects against both respiratory disorders and oral inflammatory conditions (He et al. 2024), as well as antiviral capacity, such as anti‐influenza activity (Xiao et al. 2022). Among these, the potent antioxidant properties are especially critical, as they underpin the value of Chinese olive fruit, leaves, and their extracts in traditional and modern medicine for treating chronic and degenerative diseases such as hepatitis, stomatitis, and chronic enteritis (Chang et al. 2017; Mogana and Wiart 2011). The Chinese olive is highly regarded as a medicinal resource due to its rich content of essential bioactive compounds, including polyphenols, flavonoids, and polysaccharides (Wang et al. 2022). The antioxidant capacity of plants is driven by the synergistic interaction of these bioactive compounds (Yang et al. 2023). While previous studies established that phenolic compounds, flavonoids, and polysaccharides in the Chinese olive contribute to its antioxidant capacity, the role of alkaloids remains unclear (Yu et al. 2023; Zeng et al. 2015). Further investigation is needed to explore the correlation between bioactive compounds and antioxidant capacity in Chinese olive leaves and fruits and to identify the key components for antioxidant activity. This research will provide valuable insights for optimizing bioactive compound levels to enhance the overall antioxidant capacity of the Chinese olive.

The content of bioactive compounds in different plant varieties is influenced by various factors, with genotype being a key determinant of these variations (Han et al. 2023). Additionally, the synthesis of bioactive compounds is affected by cultivation conditions, particularly the availability of mineral nutrients (Sun et al. 2021). Previous studies have shown that variations in mineral content among different cultivars can lead to differing levels of bioactive compounds, though the outcomes differ across plant species. For example, iron (Fe) was identified as the primary factor influencing total flavonoid and phenolic content among pigeon pea cultivars, while nitrogen (N) had the most significant impact on flavonoid levels in *Trifolium pratense* plants (Gerrano et al. 2022; Whittaker et al. 2009). To enhance the levels of bioactive compounds in Chinese olive through fertilization management, it is essential to investigate the relationship between essential nutrient element content and these compounds. Understanding how specific essential nutrients influence the concentration of antioxidative bioactive compounds in leaves and fruits can help optimize their levels in Chinese olive.

Over the past few decades, most studies have focused on examining genetic variations in Chinese olive fruit qualities, particularly regarding bioactive components and antioxidant activity (Chang et al. 2017; 2020). However, studies exploring the connection between bioactive compounds and antioxidant capacity in Chinese olive are limited, and research examining the relationship between essential nutrient element content and bioactive compounds is lacking (He et al. 2024; Zhang and Lin 2008). To bridge this gap, identify elite medicinal cultivars, and establish a foundation for breeding programs and fertilization optimization, we analyzed 13 representative Chinese olive cultivars harvested from the same orchard in South China. Quantitative assessments revealed substantial variation in mineral content, bioactive compound levels, and antioxidant capacity among cultivars. Pearson correlation analysis identified significant associations between mineral levels and bioactive compound content, as well as between bioactive compound content and antioxidant capacity. Multivariate analyses (principal component analysis, PCA; hierarchical cluster analysis, HCA) further classified cultivars into distinct clusters based on integrated profiles of minerals, bioactive compounds, and antioxidant capacity, elucidating cultivar‐specific metabolic signatures. These results not only contribute to the development of Chinese olive cultivars with enhanced medicinal properties but also provide valuable practical guidance for optimizing fertilization strategies in the cultivation of high‐quality medicinal Chinese olive varieties.

## 2 Materials and Methods

### 2.1 Plant Material

In December 2023, mature leaves and fruits that had reached consumption maturity were harvested from a germplasm garden in Guangdong, China, located at 23°42′6″ N, 116°53′11″ E. Thirteen cultivars—Zachi (ZC), Suanghuxiang (SHX), Fengyu1 (FY1), Langshang (LS), Lingfeng (LF), Xiaodingxiang (XDX), Tianzhong (TZ), Ma2 (M2), Fenghu (FH), Liehuo (LH), Xi1 (X1), Wangbo (WB), and Qingying (QY)—were cultivated in the orchard. Samples per cultivar were individually collected from five trees (trunk diameter: 15–25 cm). Considering the control of biological heterogeneity and the requirements of chemical quantification, we prioritized weight‐based sampling rather than quantity‐based methods. From each tree, approximately 0.5 kg of fresh leaves and 1 kg of fruit were gathered from the four cardinal directions and different heights. All selected trees received identical agronomic management practices. Fresh samples were oven‐dried at 70°C to constant weight, ground to a fine powder, and stored in sealed bags.

### 2.2 Total Carbon and Mineral Content

The total Carbon (C) and nitrogen (N) content (mg/g dry weight) in the dried samples was measured using an element analyzer (NA‐2500 Thermo Analyzer, Thermo Fisher Scientific, USA). For the determination of total phosphorus (P) content (mg/g dry weight), dried samples (0.1 g) were digested with H_2_SO_4_ and analyzed using a continuous flow analyzer (Skalar San++, Skalar Analytical, Netherlands). Total potassium (K) and calcium (Ca) content (mg/g dry weight) were measured with an atomic absorption spectrometer (Atomic Absorption Spectrophotometer, Hitachi, Japan) following a previously described method (Chen et al. 2011).

### 2.3 Bioactive Compound Content

A 0.1 g sample was extracted in a 60°C water bath using an ultrasonic extractor (CW‐2000, Xintuo, China) for 2 h with 10 mL of 60% ethanol. The mixtures were then centrifuged at 10,000 × g for 15 min at 25°C, and the supernatants were collected for further analysis. Total phenolic content (mg/100 g dry weight) was determined by the Folin–Ciocalteu method, and total flavonoids (mg/100 g dry weight) were quantified via sodium nitrite–aluminum nitrate colorimetry (Zhou et al. 2019). The alkaloid content (mg/100 g dry weight) was assessed with the bromocresol green indicator method (Fadhil et al. 2007). Total polysaccharide content was analyzed using the DNS method (Zhang et al. 2022).

### 2.4 Measurement of Antioxidant Capacity

#### 2.4.1 2,2‐Diphenyl‐1‐Picrylhydrazyl (DPPH) Radical Scavenging Ability

The DPPH radical scavenging activity (mmol TE/100 g dry weight) was assessed using a test kit from Suzhou Comin Biotechnology Co. (Suzhou, China). A 0.1 g sample was homogenized in 1 mL of extraction solution, followed by centrifugation at 10,000 × g for 10 min. The supernatants were collected and used in the DPPH radical scavenging assay, where the decolorization reaction between DPPH and antioxidants was measured, with absorbance recorded at 510 nm.

#### 2.4.2 Ferric Reducing Antioxidant Power (FRAP)

The FRAP radical scavenging activity (mmol TE/100 g dry weight) was evaluated using a test kit from Suzhou Comin Biotechnology Co. (Suzhou, China). A 0.1 g sample was homogenized in 1 mL of extraction solution and then centrifuged at 10,000 × g for 10 min. The supernatants were collected for the FRAP assay, which followed the ferric‐reducing antioxidant power method, with absorbance measured at 593 nm.

#### 2.4.3 2,2‐Azino‐Bis (3‐Ethylbenzothiazoline‐6‐Sulfonic Acid) (ABTS) Radical Scavenging Ability

The ABTS radical scavenging activity (mmol TE/100 g dry weight) was determined using a test kit from Suzhou Comin Biotechnology Co. (Suzhou, China). A 0.02 g sample was homogenized in 1 mL of extraction solution, followed by centrifugation at 10,000 × g for 10 min. The supernatants were then collected for analysis. The ABTS^+^ combined with antioxidants, triggering a decolorization reaction, and the absorbance was recorded at 734 nm.

### 2.5 Statistical Analysis

A one‐way ANOVA was conducted using SPSS 19.0 (SPSS Inc., Chicago, IL, USA) to assess differences in means, with Duncan's test employed for post hoc comparisons (*p* < 0.05). Pearson correlation analysis was used to investigate the relationships between various indices. CA, based on Euclidean distance, was performed to group samples according to their feature similarities. Additionally, principal component analysis and hierarchical cluster analysis were applied using SPSS 19.0 to visualize the similarities and differences among the test cultivars and to explore the correlations between the measured indices.

## 3 Results and Discussion

### 3.1 Essential Nutrient Element Content in Chinese Olive Leaves and Fruits

Concentrations of all analyzed macronutrients in leaves and fruits varied significantly across cultivars (Tables 1 and 2). In the leaves, the greatest variability was observed in total calcium (Ca), with a coefficient of variation (CV) of 33.80%, while total C content showed the least variation at 7.00% (Table 2). In the fruits, total P content exhibited the highest CV at 38.00%, and total C content had the lowest at 8.10% (Table 2). Generally, the leaves had higher concentrations of the most analyzed elements compared to the fruits, particularly C, N, P, and Ca. The exception was K, which was significantly more abundant in the fruits (Tables 1 and 2). N was the dominant mineral in the leaves, averaging 24.42 mg/g, followed by Ca at 11.23 mg/g, K at 7.64 mg/g, and P at 1.25 mg/g (Table 2). In contrast, K was the most abundant mineral in the fruits, averaging 27.77 mg/g, followed by N at 24.24 mg/g, Ca at 6.51 mg/g, and P at 1.11 mg/g (Table 2).

**TABLE 1:** the mineral content (mg/g) in *Canarium album* leaves and fruits varies depending on the cultivar.

Cultivar	Leaves					Fruits				
	C	N	P	K	Ca	C	N	P	K	Ca
ZC	404.05 ± 33.75^abc^	17.21 ± 0.65^ef^	1.29 ± 0.003^h^	8.02 ± 0.09^e^	8.74 ± 0.09^f^	336.97 ± 11.95^de^	33.18 ± 2.78^b^	0.65 ± 0.003^l^	35.96 ± 0.01^b^	5.53 ± 0.08^hi^
XHX	406.76 ± 12.76^abc^	14.45 ± 3.22^f^	2.00 ± 0.003^i^	6.07 ± 0.004^h^	6.15 ± 0.08^h^	376.35 ± 0.45^abcd^	11.34 ± 0.41^g^	1.39 ± 0.007^d^	21.30 ± 0.19^j^	4.69 ± 0.01^j^
FY1	415.00 ± 22.49^bc^	35.85 ± 5.04^a^	1.52 ± 0.004^b^	8.69 ± 0.011^d^	8.82 ± 0.11^f^	318.03 ± 7.51^e^	18.04 ± 1.24^fg^	0.80 ± 0.002^j^	27.50 ± 0.18^g^	6.29 ± 0.11^ef^
LS	357.37 ± 7.17^bc^	18.62 ± 2.49^ef^	1.34 ± 0.006^g^	7.36 ± 0.06^f^	14.38 ± 0.06^c^	374.80 ± 1.60^abc^	17.41 ± 0.25^fg^	1.47 ± 0.002^c^	22.72 ± 0.09^i^	6.19 ± 0.15^fg^
LF	360.65 ± 0.75^bc^	31.80 ± 1.82^abc^	0.59 ± 0.001^k^	9.94 ± 0.04^b^	16.11 ± 0.03^ab^	346.70 ± 12.33^cde^	42.96 ± 3.61^a^	0.51 ± 0.005^m^	37.20 ± 0.36^a^	6.31 ± 0.07^def^
XDX	388.05 ± 24.55^abc^	16.70 ± 1.77^ef^	1.41 ± 0.006^d^	4.82 ± 0.06^k^	8.80 ± 0.02^f^	344.2 ± 10.90^cde^	27.78 ± 1.96^bc^	1.25 ± 0.005^e^	24.71 ± 0.14^h^	8.35 ± 0.10^b^
TZ	434.45 ± 17.70^a^	32.11 ± 1.38^abc^	0.57 ± 0.001^l^	6.60 ± 0.08^g^	16.94 ± 0.28^a^	358.95 ± 12.15^abcde^	25.90 ± 2.79^cde^	0.75 ± 0.005^k^	30.84 ± 0.04^d^	6.73 ± 0.12^c^
M2	429.50 ± 26.00^a^	21.17 ± 1.16^def^	1.38 ± 0.005^e^	9.03 ± 0.05^c^	6.60 ± 0.13^gh^	394.40 ± 15.62^abc^	12.52 ± 1.23^g^	1.03 ± 0.009^g^	20.13 ± 0.10^k^	5.86 ± 0.10^gh^
FH	416.83 ± 15.55^bc^	25.81 ± 0.94^cd^	1.42 ± 0.003^c^	11.56 ± 0.05^a^	11.86 ± 0.11^d^	364.10 ± 15.50^abcde^	27.60 ± 1.60^bcd^	0.93 ± 0.006^h^	29.59 ± 0.07^e^	6.65 ± 0.09^cde^
LH	349.60 ± 12.25^c^	20.51 ± 0.83^def^	1.13 ± 0.001^j^	6.48 ± 0.04^g^	7.46 ± 0.06^g^	409.60 ± 17.10^a^	20.86 ± 0.89^def^	1.65 ± 0.011^b^	23.27 ± 0.14^i^	5.47 ± 0.10^i^
X1	400.77 ± 9.73^abc^	33.26 ± 0.73^ab^	1.56 ± 0.003^a^	5.17 ± 0.06^j^	15.28 ± 0.86^bc^	406.65 ± 20.25^ab^	28.78 ± 0.88^bc^	1.94 ± 0.024^a^	28.75 ± 0.23^f^	6.67 ± 0.06^cd^
WB	382.75 ± 11.75^abc^	27.25 ± 1.99^bcd^	1.36 ± 0.004^f^	5.61 ± 0.02^i^	14.41 ± 0.02^c^	355.00 ± 22.60^bcde^	20.4 ± 2.09^ef^	1.21 ± 0.005^f^	24.14 ± 0.08^h^	8.88 ± 0.19^a^
QY	375.97 ± 5.66^abc^	22.83 ± 1.04^de^	1.42 ± 0.006^cd^	9.84 ± 0.06^b^	10.47 ± 0.08^e^	322.63 ± 11.40^e^	28.32 ± 1.09^bc^	0.83 ± 0.005^i^	34.90 ± 0.12^c^	6.89 ± 0.04^c^

*Note:* Different letters indicate statistically significant differences (*p* < 0.05) between leaves or fruits from different cultivars.

Abbreviations: C, carbon; Ca, calcium; FH, Fenghu; FY1, Fengyu1; K, potassium; LF, Lingfeng; LH, Liehuo; LS, Langshang; M2, Ma2; N, nitrogen; P, phosphorus; QY, Qingying, SHX, Suanghuxiang, TZ, Tianzhong; WB, Wangbo; X1, Xi1; XDX, Xiaodingxiang; ZC, Zachi.

**TABLE 2:** Descriptive statistics for mineral elements, bioactive compounds and antioxidant capacity.

	Indices	Min	Max	Mean	Standard deviation (SD)	Coefficient of variations (CV, %)
Leaves	Total C content	349.60	434.45	393.98	27.51	7.0
	Total N content	14.44	35.84	24.42	7.10	29.1
	Total P content	0.56	1.56	1.24	0.31	25.5
	Total K content	4.83	11.56	7.64	2.08	27.2
	Total Ca content	6.15	16.94	11.23	3.80	33.8
	Total phenolic content	808.90	1107.12	971.62	88.24	9.1
	Total flavonoid content	230.58	373.94	290.78	42.77	14.7
	Total alkaloid content	167.70	291.79	234.34	33.96	14.5
	Total polysaccharide content	3756.72	5771.83	4601.54	700.48	15.2
	DPPH radical scavenging ability	14.69	52.13	28.63	13.34	46.9
	FRAP radical scavenging ability	11.24	36.93	20.01	8.61	43.0
	ABTS radical scavenging ability	32.48	54.28	44.66	6.40	14.3
	Total C content	318.03	409.60	362.18	29.44	8.1
	Total N content	11.33	42.96	24.24	8.71	35.9
	Total P content	0.51	1.94	1.11	0.42	38.0
	Total K content	20.13	37.20	27.77	5.69	20.5
Fruits	Total Ca content	4.70	8.88	6.51	1.13	17.3
	Total phenolic content	899.92	1266.19	1063.66	109.49	10.3
	Total flavonoid content	128.811	351.821	236.493	66.784	28.2
	Total alkaloid content	150.07	214.44	176.12	214.44	12.3
	Total polysaccharide content	2419.22	5566.62	4072.52	910.69	22.4
	DPPH radical scavenging ability	28.40	61.73	42.31	10.4	24.8
	FRAP radical scavenging ability	11.28	45.51	27.58	9.08	32.9
	ABTS radical scavenging ability	16.75	48.63	31.19	10.80	34.6

Abbreviations: C, carbon; Ca, calcium; K, potassium; N, nitrogen; P, phosphorus.

The total C content ranged from 318.03 to 434.45 mg/g, with the highest values found in the TZ cultivar's leaves and the LH cultivar's fruits (Tables 1 and 2). Total N content varied from 11.33 to 42.96 mg/g, with the FY1 leaves and LF fruits showing the highest levels (Tables 1 and 2). The total P content ranged from 0.51 to 1.94 mg/g, with the X1 cultivar's leaves and fruits exhibiting the highest levels (Tables 1 and 2). K content in Chinese olive varied from 4.83 to 37.2 mg/g, with the FH leaves and LF fruits having significantly higher K levels than other cultivars (Tables 1 and 2). The highest Ca content was found in TZ leaves and WB fruits, with Ca levels ranging from 4.70 to 16.94 mg/g (Tables 1 and 2).

Research on the essential nutrient elements in Chinese olive leaves and fruits is currently limited, especially in the leaves. Chen et al. (2021) reported variations in the essential nutrient element content among different Chinese olive fruit varieties, noting particularly high levels of Ca. Their study also identified K as the dominant mineral in Chinese olive fruits, a finding consistent with the results of the current study. This study further confirmed the high K and Ca content of the fruits. The K content averaged 27.77 mg/g (equivalent to 2777 mg per 100 g serving), covering 79% of the WHO‐recommended daily intake (3510 mg/day) for adults. Similarly, Ca levels averaged 6.51 mg/g (equivalent to 651 mg per 100 g), providing 65% of the WHO‐recommended daily intake (1000 mg/day) for individuals aged 19**–**50 years. These values highlight the potential of Chinese olive fruits as a nutrient‐dense dietary resource, particularly for populations prioritizing natural food sources to meet mineral requirements. The combined roles of K and Ca in physiological functions such as blood pressure regulation and bone metabolism (Bonewald 2019) further highlight their significance in preventive nutrition. Therefore, both leaves and fruits of Chinese olive cultivars could serve as strategic dietary components to address global deficiencies in these essential minerals.

### 3.2 Bioactive Compound Content in Chinese Olive Leaves and Fruits

**FIGURE 1:**
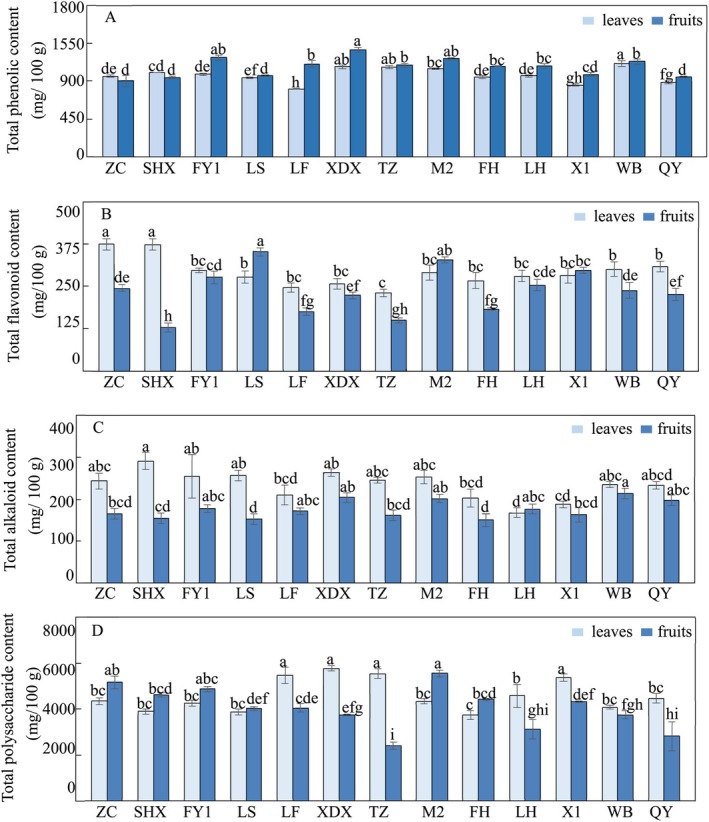
Total phenolic, flavonoid, alkaloid, and polysaccharide content in *Canarium album* leaves and fruits (A–D). Total phenolic content (A). Total flavonoid content (B). Total alkaloid content (C). Total polysaccharide content (D). Different letters imply the significant differences at *p* < 0.05 (Duncan's test). The values are expressed as the mean ± SD. FH, Fenghu; FY1, Fengyu1; LF, Lingfeng; LH, Liehuo; LS, Langshang; M2, Ma2; QY, Qingying; SHX, Suanghuxiang; TZ, Tianzhong; WB, Wangbo; X1, Xi1; XDX, Xiaodingxiang; ZC, Zachi.

The content of bioactive compounds in Chinese olive varied significantly across different cultivars (Figure 1, Table 2). In the leaves, total polysaccharide content showed the highest CV at 15.2%, followed by total flavonoid content at 14.7%, total alkaloid content at 14.5%, and total phenolic content at 9.1% (Table 2). In the fruits, total flavonoid content had the highest CV at 28.2%, followed by total polysaccharide content at 22.4%, total alkaloid content at 12.3%, and total phenolic content at 10.3% (Table 2). The leaves generally contained higher concentrations of most bioactive compounds compared to the fruits, specifically phenolics, alkaloids, and polysaccharides (Figure 1, Table 2). This may reflect the primary role of leaves in photosynthesis and stress resistance, requiring higher levels of protective compounds (Mittler 2002). The only exception was flavonoids, which were significantly more abundant in the fruits (Figure 1, Table 2). The differential accumulation of bioactive compounds in Chinese olive leaves and fruits underscores the complexity of secondary metabolism, which is influenced by a multitude of factors, including developmental stage and environmental factors (Winkel 2002). While this study focused on variability within a single garden, it should be noted that regional environmental factors (e.g., altitudinal gradients, microclimate variations) may induce differential expression of bioactive compounds in gardens located elsewhere. Nevertheless, preliminary comparisons with regional datasets indicate robust core metabolic pathways in Chinese olive cultivars (Yu et al. 2023). Our findings retain significant representativeness, particularly within Guangdong Province's horticultural contexts.

The total phenolic content in Chinese olive, ranging from 808.90 to 1266.19 mg/100 g with peak levels in WB leaves and XDX fruits, was consistent with He et al. (2024) and Chang et al. (2017) for the 13 cultivars measured. However, it was significantly lower than the values reported by Zhang and Lin (2008), likely due to the different analytical methods used—Prussian blue method versus the Folin–Ciocalteu method—which are known to significantly impact phenolic content. The total flavonoid content, varying between 128.81 and 373.94 mg/100 g with the highest in ZC leaves and LS fruits, aligned with He et al. (2024) but was higher than Chang et al. (2017). Since our study and the aforementioned studies used the sodium nitrite‐aluminum nitrate colorimetric assay, the discrepancies observed may be attributed to variations in the main cultivars and the cultivation conditions specific to Guangdong and Fujian (Macit 2021; Mpofu et al. 2006). Our study is the first to report the total alkaloid content in Chinese olive, ranging from 150.07 to 291.79 mg/100 g, with the highest in SHX leaves and WB fruits, providing a new baseline for future research. The total polysaccharide content, ranging from 2419.22 to 5771.83 mg/100 g and highest in XDX leaves and M2 fruits, is consistent with Zeng et al. (2015). These findings not only highlight the influence of extraction and determination methods on bioactive substance concentrations but also underscore significant regional differences in bioactive substance concentrations among main cultivars of Chinese olive grown in Guangdong and Fujian, which could be attributed to the unique environmental and agricultural practices of these regions.

### 3.3 Relationship Between Essential Nutrient Elements and Bioactive Compound Content

Over time, plant secondary metabolism has evolved as an adaptation to diverse ecological environments (Wang et al. 2022). This evolutionary process has resulted in the production of various bioactive compounds, such as phenolics, flavonoids, alkaloids, and polysaccharides, which play a crucial role in plant defense against environmental stress (Sharma et al. 2022). The production of these bioactive compounds is closely linked to the nutrient elements present in plants (Macit 2021; Poonia et al. 2022). For instance, Gerrano et al. (2022) identified a significant positive correlation between Fe levels and phenolic content in immature pigeon pea pods. Similarly, a strong correlation was observed between phenolic and copper (Cu) content in oat seeds (Poonia et al. 2022).

In this study, PCA was used to explore the relationship between essential nutrient elements and bioactive compound content in Chinese olive (Tables 3 and 4). In the leaves, total flavonoid content showed a significant negative correlation with both total N and Ca levels (Table 3, *p* < 0.05). This negative correlation suggests that as N and Ca levels decrease, the total flavonoid content in the leaves tends to increase. This finding supports both the resource availability hypothesis and the carbon–nutrient balance hypothesis, which propose that plants may allocate more resources to producing secondary metabolites under nutrient‐deficient conditions as a defense strategy (Coley et al. 1985; Stamp 2003).

**TABLE 3:** Pearson correlation coefficients between the mineral element contents and bioactive compound contents in leaves.

Indices	Total phenolic content	Total flavonoid content	Total alkaloid content	Total polysaccharide content
C	0.408	0.026	0.37	−0.016
N	−0.264	−0.543*	−0.382	0.321
P	0.077	0.414	0.056	−0.441
K	−0.425	−0.072	−0.155	−0.356
Ca	−0.264	−0.560*	−0.297	0.371

* Indicates significance at *p* < 0.05.

**TABLE 4:** Pearson correlation coefficients between the mineral element contents and bioactive compound contents in fruits.

Indices	Total phenolic content	Total flavonoid content	Total alkaloid content	Total polysaccharide content
C	−0.092	0.262	−0.225	0.068
N	−0.068	−0.286	−0.083	−0.233
P	−0.121	0.374	−0.079	−0.049
K	−0.303	−0.335	−0.168	−0.206
Ca	0.523*	0.038	0.625*	−0.349

* Indicates significance at *p* < 0.05.

Conversely, a significant positive correlation was found between total Ca levels in the fruits and both the total phenolic content and the alkaloid content (Table 4, *p* < 0.05). This aligns with previous research indicating that increased Ca levels can enhance the accumulation of bioactive compounds in various plants, such as buckwheat and rice (Choe et al. 2021; Sim et al. 2020). The positive correlation suggests that higher Ca levels in the fruits are associated with increased synthesis of alkaloids, possibly due to calcium's role in signaling pathways that regulate the production of bioactive compounds (Sim et al. 2020; Zhang et al. 2021). Ca was the only essential nutrient element that showed a significant correlation with both total phenolic and alkaloid content in Chinese olive fruits (Table 4). This suggests that Ca could be particularly important in regulating the production of bioactive compounds in these fruits. These findings highlight the complex relationships between nutrients and bioactive compounds in plants, where different nutrients may have varying effects depending on the tissue type and the metabolic pathways involved.

### 3.4 Antioxidant Capacity of Chinese Olive Leaves and Fruits

The antioxidant capacity of Chinese olive leaves and fruits from various cultivars was assessed using the DPPH, ABTS, and FRAP bioassays, which are standard methods for evaluating the antioxidant potential of plants (Devanesan et al. 2023; Wei et al. 2022). Significant variation in antioxidant capacity was observed among the 13 cultivars across all three assays, consistent with previous studies (Chang et al. 2017). The DPPH showed the highest variability with a CV of 46.9% in leaves, whereas in fruits, the ABTS exhibited the greatest variability, with a CV of 34.6% (Table 2).

**FIGURE 2:**
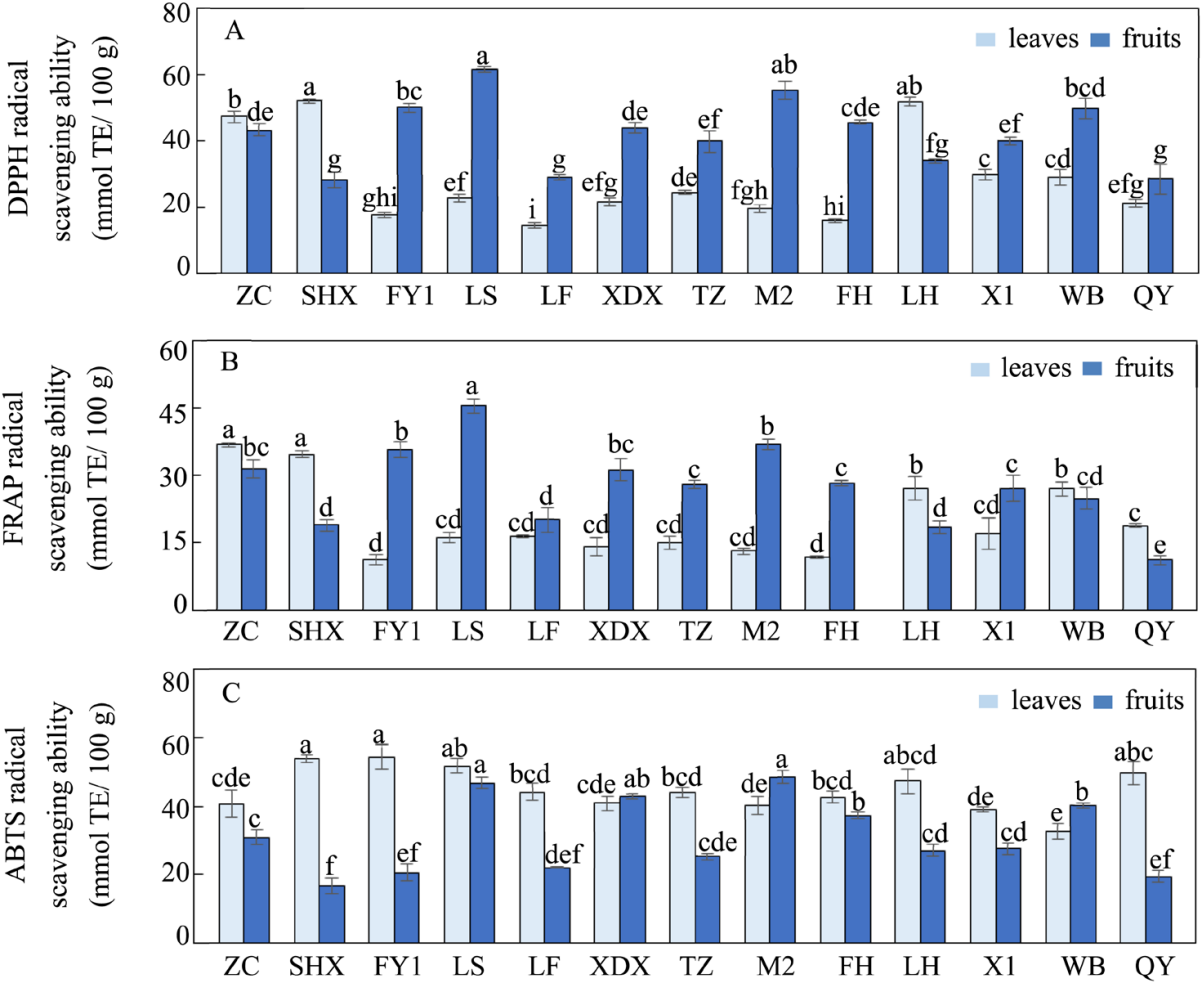
Antioxidant activity in *Canarium album* leaves and fruits. (A) DPPH radical scavenging ability. (B) FRAP radical scavenging ability. (C) ABTS radical scavenging ability. Different letters imply the significant differences at *p* < 0.05 (Duncan's test). The values are expressed as the mean ± SD. FH, Fenghu; FY1, Fengyu1; LF, Lingfeng; LH, Liehuo; LS, Langshang; M2, Ma2; QY, Qingying; SHX, Suanghuxiang; TZ, Tianzhong; WB, Wangbo; X1, Xi1; XDX, Xiaodingxiang; ZC: Zachi.

The DPPH antioxidant activity of Chinese olive ranged from 14.69 to 61.73 mmol TE/100 g, with the highest levels observed in SHX leaves and LS fruits (Figure 2, Table 2). The FRAP varied between 11.24 and 45.51 mmol TE/100 g, with ZC leaves and SHX fruits showing the highest activity (Figure 2, Table 2). The ABTS antioxidant activity ranged from 16.75 to 48.63 mmol TE/100 g, with FY1 leaves and M2 fruits exhibiting the highest levels (Figure 2, Table 2). These results highlight the wide range of antioxidant activities among different Chinese olive cultivars, underscoring the importance of selecting the appropriate cultivar to optimize their medicinal qualities.

### 3.5 Relationship Between Bioactive Compounds Content and Antioxidant Capacity

A principal component analysis (PCA) was conducted to elucidate the relationship between the content of bioactive compounds, including phenolics and polysaccharides, and antioxidant capacity in Chinese olive leaves and fruits (Tables 5 and 6). Our findings revealed no significant correlation between the total phenolic content and radical scavenging activities, as evaluated by the DPPH, FRAP, and ABTS assays in both leaves and fruits (Tables 5 and 6). This observation contrasts with previous research that has highlighted the significant role of phenolics in the antioxidant activity of Chinese olive fruits (Chang et al. 2017; He et al. 2024). Similarly, we identified no significant correlation between the total polysaccharide content and antioxidant capacity in Chinese olive leaves and fruits (Tables 5 and 6), which diverges from earlier studies suggesting that polysaccharides significantly contribute to the antioxidant properties of fruits (Rubio‐Senent et al. 2015; Zeng et al. 2015). These discrepancies may be attributed to variations in the Chinese olive varieties studied and the methods used to quantify phenolic and polysaccharide content, which could influence the results and highlight the need for standardized protocols in future research.

**TABLE 5:** Pearson correlation coefficients between the bioactive compound contents and antioxidant capacity in leaves.

Indices	DPPH radical scavenging ability	FRAP radical scavenging ability	ABTS radical scavenging ability
Total phenolic content	0.121	0.096	−0.284
Total flavonoid content	0.633*	0.750*	0.273
Total alkaloid content	−0.022	0.112	0.296
Total polysaccharide content	−0.208	−0.287	−0.281

* Indicates significance at *p* < 0.05.

**TABLE 6:** Pearson correlation coefficients between the bioactive compound contents and antioxidant capacity in fruits.

Indices	DPPH radical scavenging ability	FRAP radical scavenging ability	ABTS radical scavenging ability
Total phenolic content	0.274	0.216	0.353
Total flavonoid content	0.707**	0.621*	0.571*
Total alkaloid content	0.089	−0.159	0.284
Total polysaccharide content	0.397	0.476	0.266

Among the bioactive compounds analyzed, only total flavonoid content demonstrated a significant positive association with the radical scavenging activities of DPPH, FRAP, and ABTS in both leaves and fruits (*p* < 0.05), except for ABTS in leaves. These findings suggest that flavonoids are likely the primary contributors to antioxidant activity, which is consistent with previous studies on Chinese olive and other plants (Kuo et al. 2015; Xiang et al. 2020). The results underscore the need for further investigation into the specific mechanisms underlying antioxidant properties in Chinese olive leaves and fruits, as well as the potential variations between these tissues. This knowledge will be crucial for developing Chinese olive cultivars with enhanced medicinal qualities.

### 3.6 Evaluation of Characteristic Diversity in Chinese Olive Cultivars

**FIGURE 3:**
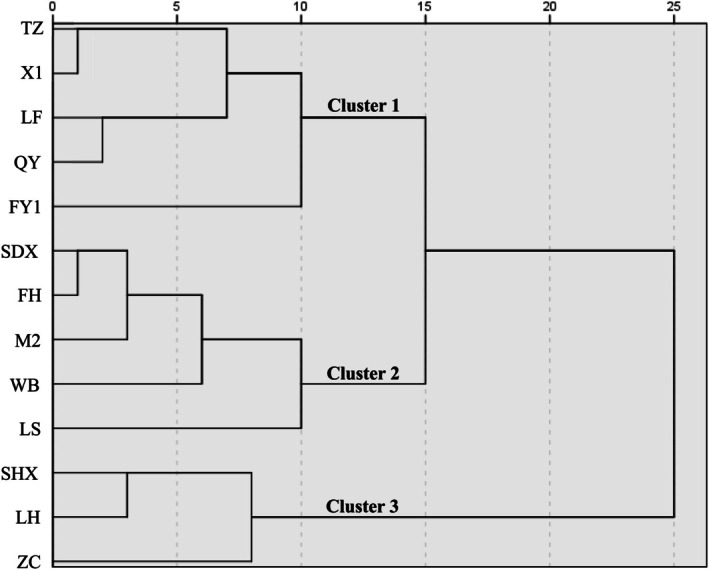
Hierarchical cluster analysis of Chinese olive based on the content of minerals, bioactive compounds, and antioxidant capacity in leaves and fruits: FH, Fenghu; FY1, Fengyu1; LF, Lingfeng; LH, Liehuo; LS, Langshang; M2, Ma2; QY, Qingying, TZ, Tianzhong; WB, Wangbo; X1, Xi1; XDX, Xiaodingxiang, ZC, Zachi.

To access the overall variation among Chinese olive cultivars, HCA and PCA were conducted. Based on the essential nutrient elements, bioactive compound contents, and antioxidant capacity in the leaves and fruits of Chinese olive, the 13 cultivars were categorized into three distinct groups (Figure 3), highlighting the significant genetic diversity among them. Cluster 1 comprised five cultivars (TZ, X1, LF, QY, FY1) characterized by the highest mean FRAP values in leaves and ABTS values in fruits (Table S1). Cluster 2 included five cultivars (XDX, FH, M2, WB, LS), which exhibited the highest mean DPPH and FRAP values in leaves (Table S1). Cluster 3 consisted of three cultivars (XHX, LH, ZC), noted for their highest mean total carbon content and FRAP values in leaves (Table S1).

**FIGURE 4:**
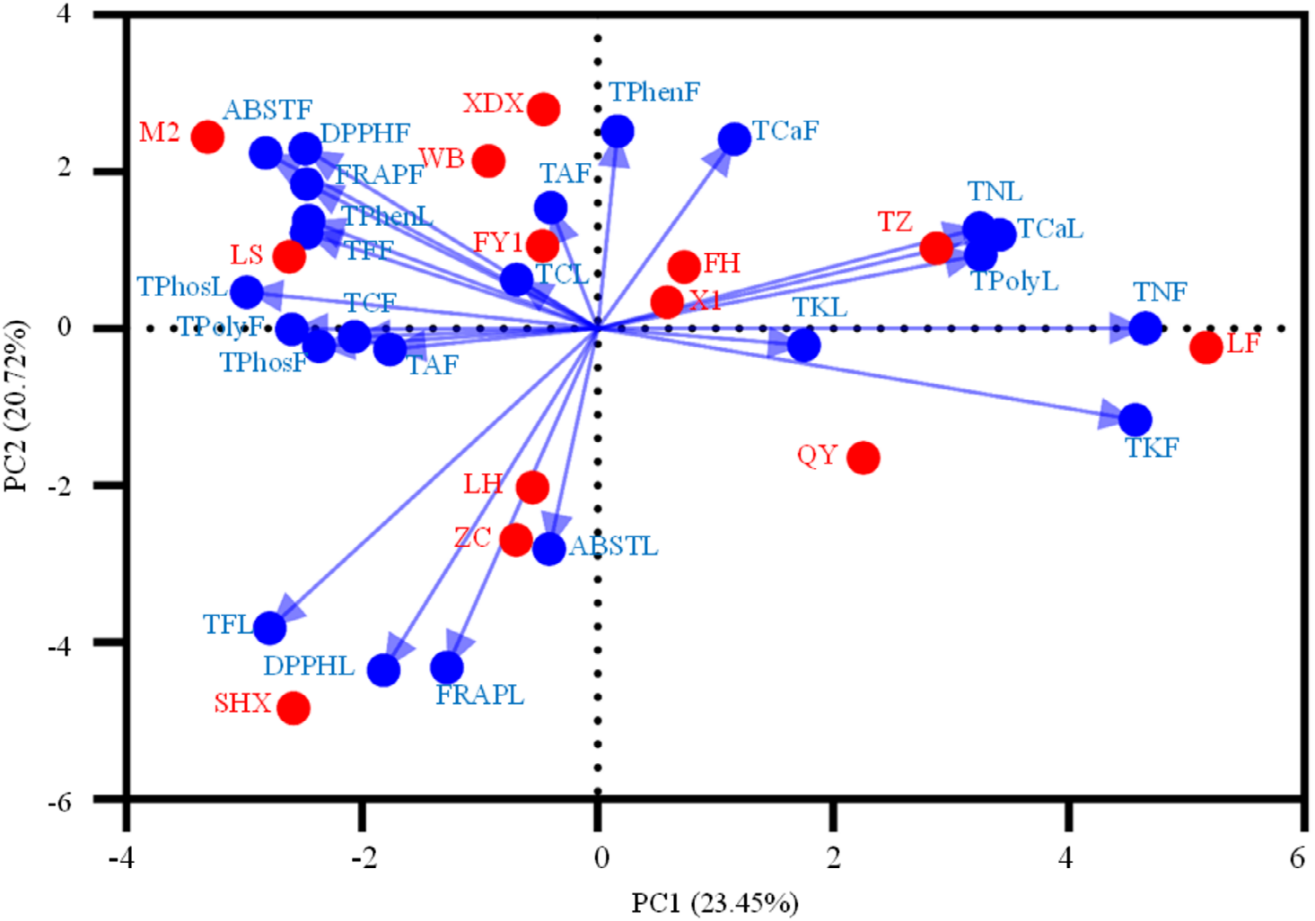
Principal component analysis (PCA) of 13 *Canarium album* cultivars based on mineral element, bioactive compounds, and antioxidant capacity. ABSTL/F, ABST radical scavenging ability in leaves/in fruits; DPPH radical scavenging ability in leaves/in fruits; FH, Fenghu; FRAPL/F, FRAP radical scavenging ability in leaves/in fruits. FY1, Fengyu1; LF, Lingfeng; LH, Liehuo; LS, Langshang; M2, Ma2; QY, Qingying, SHX, Suanghuxiang; TAL/F, total alkaloid content in leaves/fruits; DPPHL/F, TCaL/F, total calcium content in leaves/fruits; TCL/F, total carbon content in leaves/fruits; TFL/F, total flavonoid content in leaves/fruits; TKL/F, total potassium content in leaves/fruits; TNL/F, total nitrogen content in leaves/fruits; TPhenL/F, total phenolic content in leaves/fruits; TPhosL/F, total phosphate content in leaves/fruits; TPolyL/F, total polysaccharide content in leaves/fruits; TZ, Tianzhong; WB, Wangbo; X1, Xi1; XDX, Xiaodingxiang; ZC, Zachi.

The PCA model accounted for 44.17% of the total variance, with PC1 explaining 23.45% and PC2 contributing 20.72% (Figure 4). Total P, K, and Ca content were the primary variables associated with PC1, while total phenolic content, total flavonoid content, DPPH radical scavenging ability, FRAP, and ABTS radical scavenging ability were the main contributors to PC2 (Table S2). Strong correlations were found between antioxidant capacities and total flavonoid contents in the leaves of SHX, ZC, and LH cultivars (Figure 4). In fruits, these properties were closely linked to the FY1, WB, MZ, LS, and XD cultivars (Figure 4). These findings align with those from Tables 3 and 4, demonstrating a strong positive correlation between total flavonoid content and the radical scavenging activities measured by DPPH, FRAP, and ABTS in both leaves and fruits (Figure 4). The CA and PCA results indicated that the SHX, LH, and ZC cultivars possessed high overall medicinal properties in their leaves, while the XDX, WB, FY1, M2, and LS cultivars showed high overall medicinal properties in their fruits (Figures 3 and 4). Conversely, the FH, X1, TZ, LF, and QY cultivars displayed the lowest overall medicinal properties (Figure 4). Additionally, the FH, X1, and TZ cultivars consistently exhibited elevated calcium content in both leaves and fruits compared to other cultivars, making them potential candidates for calcium‐fortified supplements. Nevertheless, their overall medicinal potential remains limited due to low levels of flavonoids, phenolics, and antioxidant capacity.

PCA results revealed a strong negative correlation between the total N and K contents and the bioactive compound contents and antioxidant capacities (Figure 4). These findings, when considered alongside the data from Tables 5 and 6, suggest that deficiencies in N and K can significantly stimulate the production of bioactive compounds in Chinese olive leaves and fruits, leading to an enhanced antioxidant capacity. To maximize the medicinal potential of Chinese olive, integrating superior germplasm resources with strategically and scientifically informed fertilization practices is crucial. This approach can optimize the synthesis of bioactive compounds, thereby enhancing the plant's overall medicinal properties.

## 4 Conclusion

This study characterized variations in essential nutrients, bioactive compounds, and antioxidant capacity among Chinese olive cultivars in both leaves and fruits. In leaves, it was found that the total flavonoid content was significantly negatively correlated with total N and Ca levels. In fruits, a significant positive correlation was identified between total Ca levels and both the total phenolic content and alkaloid content. Additionally, flavonoids were found to be the dominant drivers of antioxidant activity. Among the cultivars tested: Leaves of Suanghuxiang (SHX), Liehuo (LH), and Zachi (ZC) exhibited the highest total flavonoid content and antioxidant activity; fruits of Xiaodingxiang (XDX), Wangbo (WB), Fengyu1 (FY1), Ma2 (M2), and Langshang (LS) showed the highest levels of these properties; in contrast, cultivars FH (Fenghu), X1 (Xi1), TZ (Tianzhong), LF (Lingfeng), and QY (Qingying) demonstrated the lowest overall medicinal properties, characterized by the lowest levels of total flavonoid content and antioxidant activity. These findings facilitate the breeding of high‐medicinal‐value cultivars and provide a theoretical framework for optimizing fertilization to enhance Chinese olive's medicinal value.

## Author Contributions

**Qingli Zhuang:** conceptualization (lead), data curation (lead), formal analysis (lead), investigation (lead), methodology (lead), project administration (lead), resources (lead), software (lead), supervision (lead), validation (lead), visualization (lead), writing – original draft (lead), writing – review and editing (lead). **Xin Tan:** formal analysis (lead), investigation (lead), methodology (equal). **Peiwen Ma:** methodology (equal), project administration (equal), supervision (equal), writing – original draft (equal), writing – review and editing (equal). **Duo Lai:** methodology (equal), project administration (equal), supervision (equal), visualization (equal), writing – original draft (equal), writing – review and editing (equal). **Xuehua Shao:** formal analysis (equal), writing – original draft (equal). **Ruilian Lai:** formal analysis (equal). **Weiqiang Xiao:** conceptualization (lead), data curation (lead), formal analysis (lead), funding acquisition (lead), investigation (lead), methodology (lead), project administration (lead), resources (lead), software (lead), supervision (lead), validation (lead), visualization (lead), writing – original draft (lead), writing – review and editing (lead).

## Ethics Statement

The authors have nothing to report.

## Conflicts of Interest

The authors declare no conflicts of interest.

## Supporting information


**Table S1** Cluster means for bioactive compounds, mineral elements and antioxidant capacity of 13 *Canarium album* cultivars.
**Table S2:** Eigenvalues of the first two principal components.

## Data Availability

Data Availability StatementThe data that support the findings of this study are available from the corresponding author upon reasonable request.
