# Supplementary material for: Assessment of Essential Nutrients, Bioactive Compounds, and Antioxidant Activity in the Leaves and Fruits of Chinese Olive ( Canarium album (Lour.) DC.) Cultivars
Source: Food Sci Nutr. 2025 Oct 24;13(10):e71049. doi: 10.1002/fsn3.71049 (PMC12550265; doi:10.1002/fsn3.71049)
Supplement: Supplementary file 1 — Table S1 Cluster means for bioactive compounds, mineral elements and antioxidant capacity of 13 Canarium album cultivars. Table S2: Eigenvalues of the first two principal components. [file FSN3-13-e71049-s001.docx]

**Assessment of** **essential nutrients, bioactive compounds, and antioxidant activity in the leaves and fruits of Chinese olive (*Canarium album*) cultivars**

Qingli Zhuang^1^ | Xin Tan^1^ | Peiwen Ma^2^ | Duo Lai^1^ | Xuehua Shao^1^ | Ruilian Lai^3^ | Weiqiang Xiao^1^

*^1^Institute of Fruit Tree Research,* *Guangdong Academy of Agricultural Sciences; Key Laboratory of South Subtropical Fruit Biology and Genetic Resource Utilization, Ministry of Agriculture and Rural Affairs; Guangdong Provincial Key Laboratory of Science and Technology Research on Fruit Tree, Guangzhou, 510640, China.*

*^2^Department of Gastroenterology, Beijing Puren Hospital, Beijing 100062, China*

*^3^Fruit Research Institute, Fujian Academy of Agricultural Sciences, Fuzhou, Fujian 350013, China*

**Correspondence:** Weiqiang Xiao (xwq6817@126.com)

**Index of the supllementary data**

**Table S1:** Cluster means for bioactive compounds, mineral elements and antioxidant capacity of 13 *Canarium album* cultivars.

**Table S2:** Eigenvalues of the first two principal components.

| Indices | Cluster 1 | Cluster 2 | Cluster 3 |
| --- | --- | --- | --- |
| Total carbon content in leaves | 357.37 | 406.77 | 360.65 |
| Total nitrogen content in leaves | 18.62 | 14.45 | 31.80 |
| Total phosphorus content in leaves | 1.34 | 1.20 | 0.59 |
| Total kalium content in leaves | 0.74 | 0.61 | 0.99 |
| Total calcium content in leaves | 1.44 | 0.61 | 1.61 |
| Total phenolic content in leaves | 9.41 | 10.02 | 8.09 |
| Total flavonoid content in leaves | 3.04 | 3.73 | 2.45 |
| Total alkaloid content in leaves | 2.56 | 2.92 | 2.10 |
| Total polysaccharide content in leaves | 38.77 | 39.04 | 54.74 |
| DPPH radical scavenging ability in leaves | 227.39 | 521.40 | 146.99 |
| FRAP radical scavenging ability in leaves | 516.19 | 537.92 | 440.80 |
| ABTS radical scavenging ability in leaves | 374.80 | 376.35 | 346.70 |
| Total carbon content in fruits | 17.42 | 11.34 | 42.96 |
| Total nitrogen content in fruits | 1.47 | 1.39 | 0.51 |
| Total phosphorus content in fruits | 2.27 | 2.13 | 3.72 |
| Total kalium content in fruits | 0.62 | 0.47 | 0.63 |
| Total calcium content in fruits | 9.64 | 9.42 | 11.04 |
| Total phenolic content in fruits | 3.52 | 1.29 | 1.76 |
| Total flavonoid content in fruits | 1.52 | 1.55 | 1.72 |
| Total alkaloid content in fruits | 162.92 | 347.02 | 165.15 |
| Total polysaccharide content in fruits | 40.26 | 46.18 | 40.51 |
| DPPH radical scavenging ability in fruits | 617.36 | 284.08 | 291.18 |
| FRAP radical scavenging ability in fruits | 455.15 | 189.82 | 201.71 |
| ABTS radical scavenging ability in fruits | 468.14 | 167.53 | 221.57 |

**Table S1** | Cluster means for bioactive compounds, mineral elements and antioxidant capacity of 13 *Canarium album* cultivars.

**Table S2** **|** Eigenvalues of the first two principal components.

| Indices | PCA 1 | PCA 2 |
| --- | --- | --- |
| Total carbon content in leaves | -0.14 | 0.184 |
| Total nitrogen content in leaves | 0.581 | 0.377 |
| Total phosphorus content in leaves | -0.606 | 0.136 |
| Total kalium content in leaves | 0.314 | -0.037 |
| Total calcium content in leaves | 0.611 | 0.352 |
| Total phenolic content in leaves | -0.502 | 0.359 |
| Total flavonoid content in leaves | -0.566 | -0.659 |
| Total alkaloid content in leaves | -0.420 | -0.018 |
| Total polysaccharide content in leaves | 0.582 | 0.276 |
| DPPH radical scavenging ability in leaves | -0.369 | -0.751 |
| FRAP radical scavenging ability in leaves | -0.260 | -0.745 |
| ABTS radical scavenging ability in leaves | -0.084 | -0.485 |
| Total carbon content in fruits | -0.358 | -0.046 |
| Total nitrogen content in fruits | 0.833 | 0.001 |
| Total phosphorus content in fruits | -0.481 | -0.039 |
| Total kalium content in fruits | 0.817 | -0.201 |
| Total calcium content in fruits | 0.208 | 0.712 |
| Total phenolic content in fruits | 0.030 | 0.742 |
| Total flavonoid content in fruits | -0.499 | 0.406 |
| Total alkaloid content in fruits | -0.081 | 0.454 |
| Total polysaccharide content in fruits | -0.528 | -0.002 |
| DPPH radical scavenging ability in fruits | -0.573 | 0.660 |
| FRAP radical scavenging ability in fruits | -0.502 | 0.543 |
| ABTS radical scavenging ability in fruits | -0.504 | 0.676 |
